# Supplementary material for: Cholinergic basal forebrain neurons regulate vascular dynamics and cerebrospinal fluid flux
Source: Nat Commun. 2025 Jun 23;16:5343. doi: 10.1038/s41467-025-60812-3 (PMC12185694; doi:10.1038/s41467-025-60812-3)
Supplement: Supplementary file 1 — Supplementary Information [file 41467_2025_60812_MOESM1_ESM.pdf]

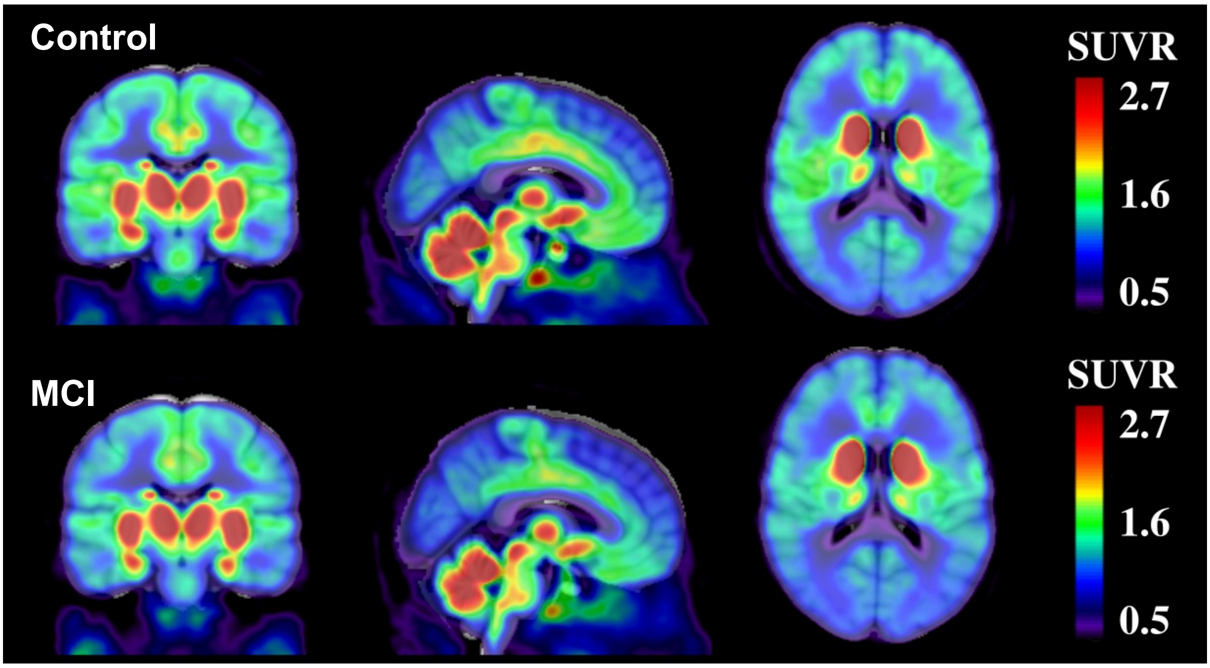

Supplementary Fig. 1. FEOBV in aged human subjects. The averaged FEOBV SUVR images of the control (n = 12, upper row) and MCI (n = 9, bottom row) groups.

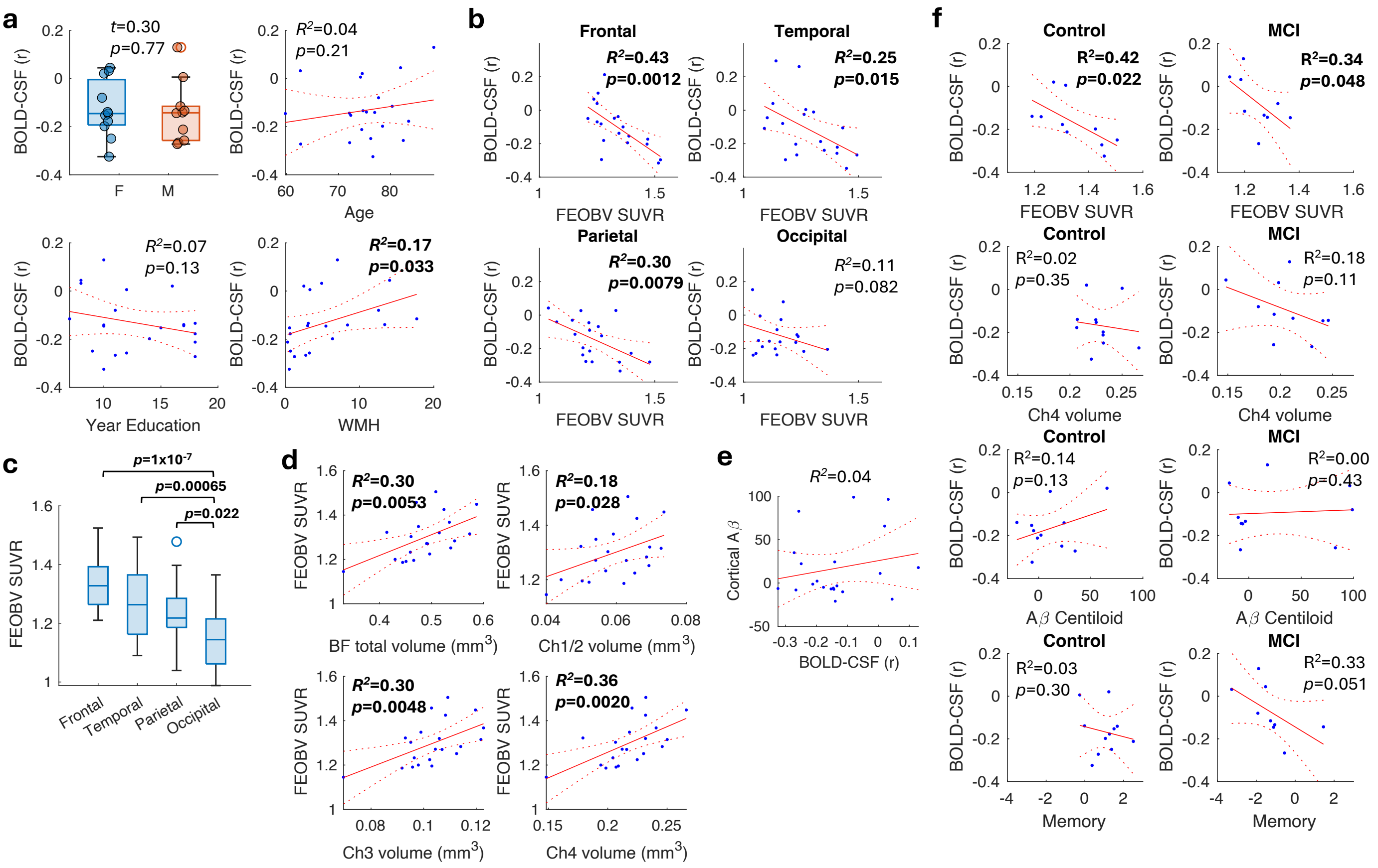

Supplementary Fig. 2. BOLD-CSF coupling in aged human subjects. **a)** The cortical BOLD-CSF coupling did not differ by sex (n = 10 males and 11 females; t-Test, two-tailed), age (n = 21; one-tailed) or year of education (n = 21; one-tailed). However, it correlated (one-tailed) with the volume of white matter hyperintensities (WMH), an indication of small vessel disease. **b)** The regional BOLD-CSF coupling correlated with regional FEOBV SUVR in the frontal, parietal and temporal lobes (n = 19; one-tailed, uncorrected). **c)** The regional FEOBV SUVR (n = 21) was significantly lower in the occipital lobe compared to other cortical regions (one-way ANOVA). **d)** The cortical FEOBV SUVR correlated with the basal forebrain (BF) total and subregional volumes, particularly the Ch4 region (n = 21; one-tailed, uncorrected). **e)** The cortical BOLD-CSF coupling did not correlate with the cortical Aβ Centiloid scale (n = 21). **f)** Correlation between BOLD-CSF coupling and (from top to bottom) FEOBV, Ch4 volume, cortical amyloid burden, and memory performance in control (n = 10; left) and MCI (n = 9; right) subjects separately (one-tailed, uncorrected).

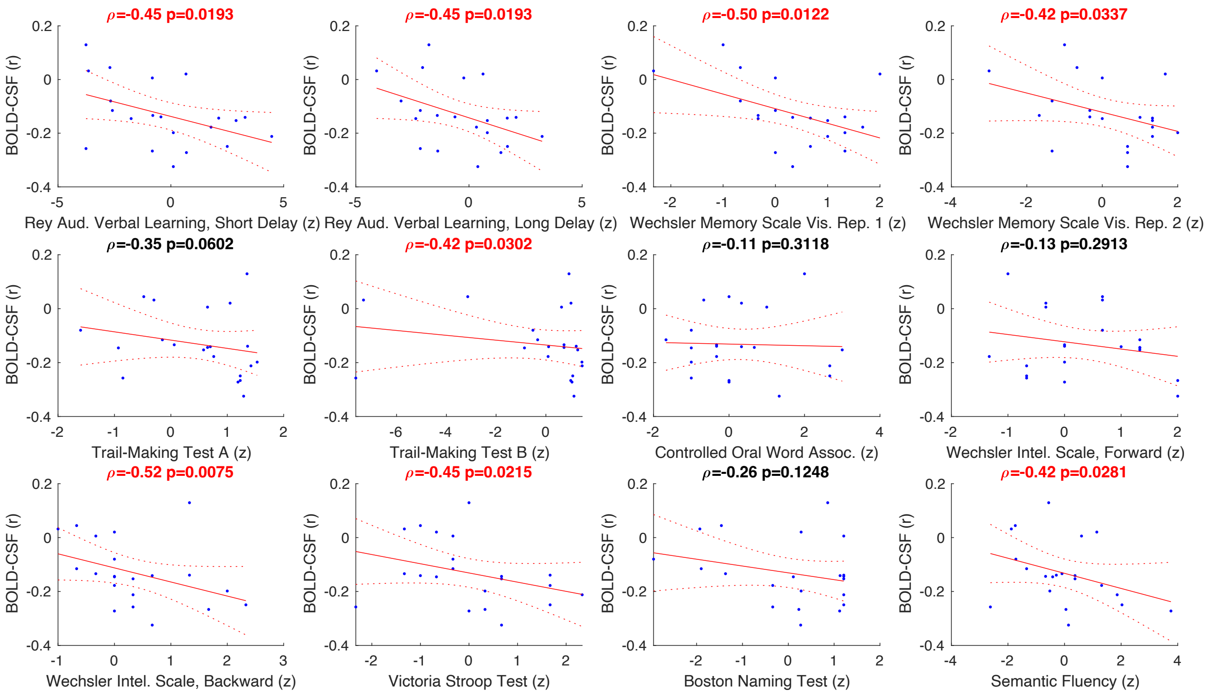

Supplementary Fig. 3. Relationship between BOLD-CSF coupling and neurocognitive assessment. The cortical BOLD-CSF coupling correlated with a battery of neuropsychological assessments (n = 21; Spearman's correlation, two-tailed, uncorrected). The Rey Auditory Verbal Learning Test – short delay & long delay, Wechsler Memory Scale - Visual Reproduction 1 & 2 were used to assess memory. The Trail-Making Test B, Controlled Oral Word Association Test, and Wechsler Adult Intelligence Scale - Digit Span (Backwards) were used to assess executive function. The Trail-Making Test A and Victoria Stroop Test were used to assess attention. The Boston Naming Test and Semantic Fluency Test were used to assess language. It should be noted that in these exploratory analyses, the p-values were not adjusted for multiple testing.

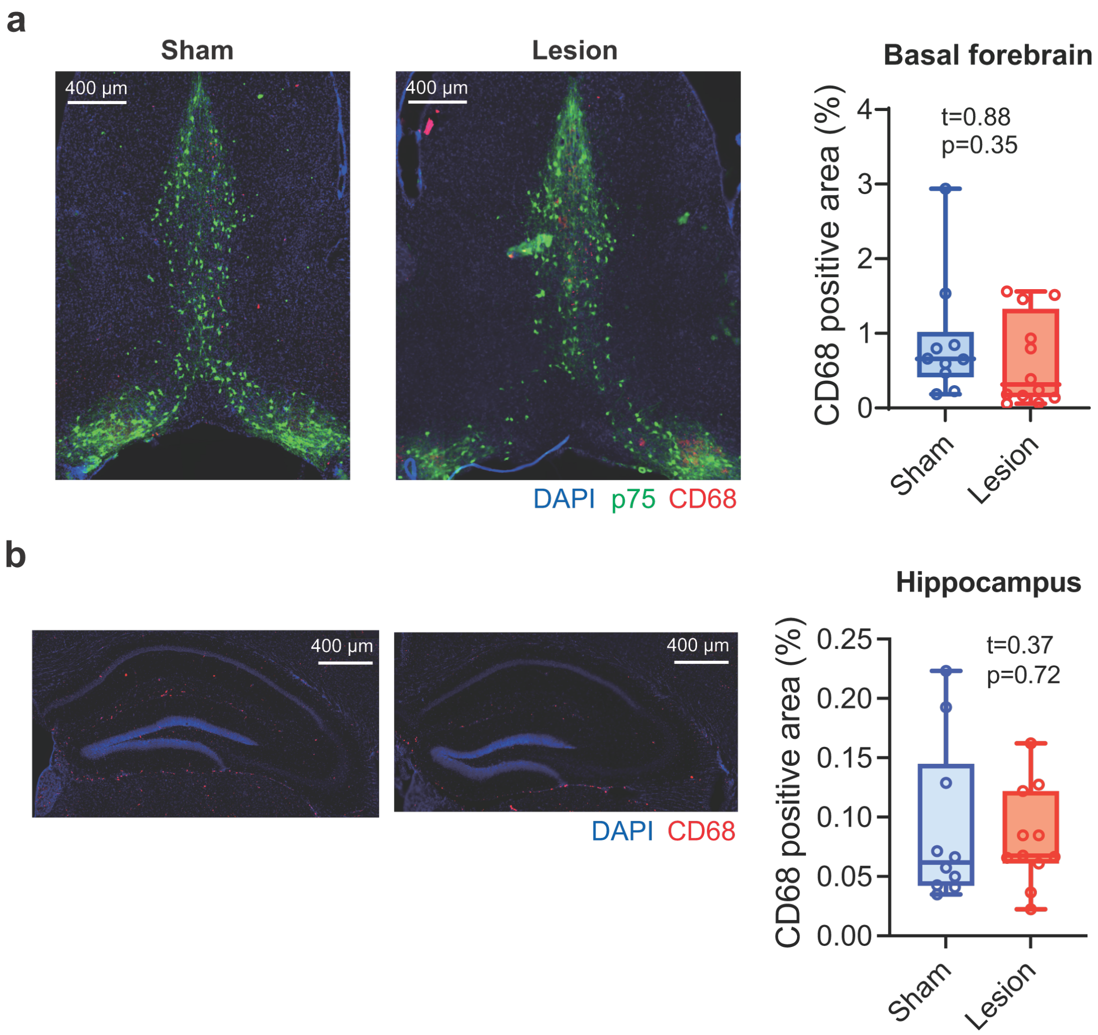

Supplementary Fig. 4. CD68 staining of reactive microglia. No difference in CD68 positive area was found between Sham and Lesion groups in the basal forebrain (n = 10 Sham and 12 Lesion) and hippocampus (n = 10 Sham and 11 Lesion) (two-sample t-Test, two tailed). The CD68 fluorescent signal intensity was thresholded to remove the background. The CD68 positive area (%) = CD68 positive area / Total ROI area *100%.

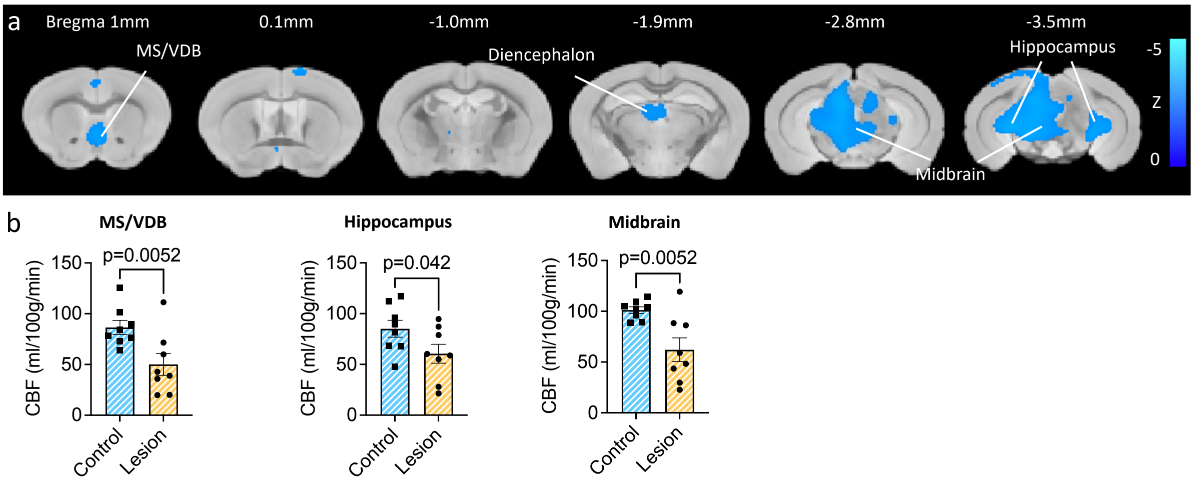

Supplementary Fig. 5. CBF is reduced in cholinergic-lesioned mice. a) Voxel-wise comparison of the cerebral blood flow (CBF), measured by arterial spin labelling MRI, between the cholinergic-lesioned mice (n = 8) and control (n = 8; two-sample t-test, FDR-corrected). Color map overlaid on the brain template indicates change of z-score (blue represents reduction in the lesioned mice). **b**) ROI analysis shows highly reduced CBF in the MS/VDB and midbrain, and moderately reduced CBF in the hippocampus (n=8 per group, two-sample t-test).

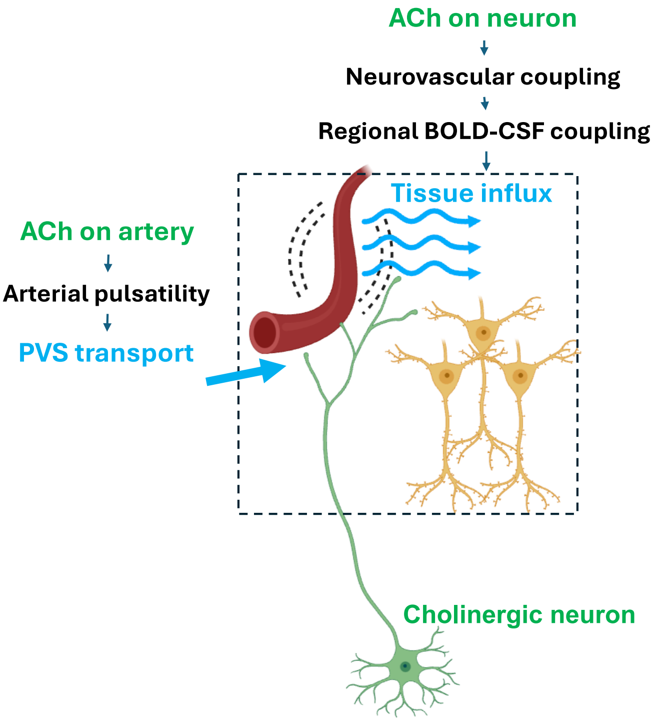

Supplementary Fig. 6. A working model of BFCN regulation of fluid flux. The BFCN innervation on cerebral arteries modulates arterial pulsation, leading to a change in perivascular fluid transport. The BFCN innervation on neurons modulates the coherent activity in brain tissue, leading to a change in regional BOLD signal oscillation via neurovascular coupling. Concorded regional BOLD and CSF oscillations facilitate fluid influx into the brain region. ACh: acetylcholine. The diagram was created in BioRender. Chuang, K. (2025) https://BioRender.com/09f0bqr.

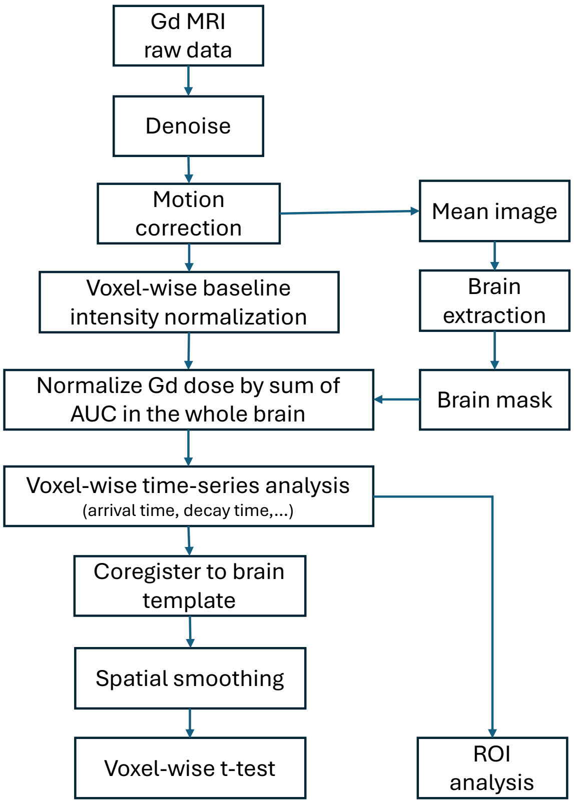

Supplementary Fig. 7. The data processing workflow for the intracisternal Gd-based contrast enhanced MRI.

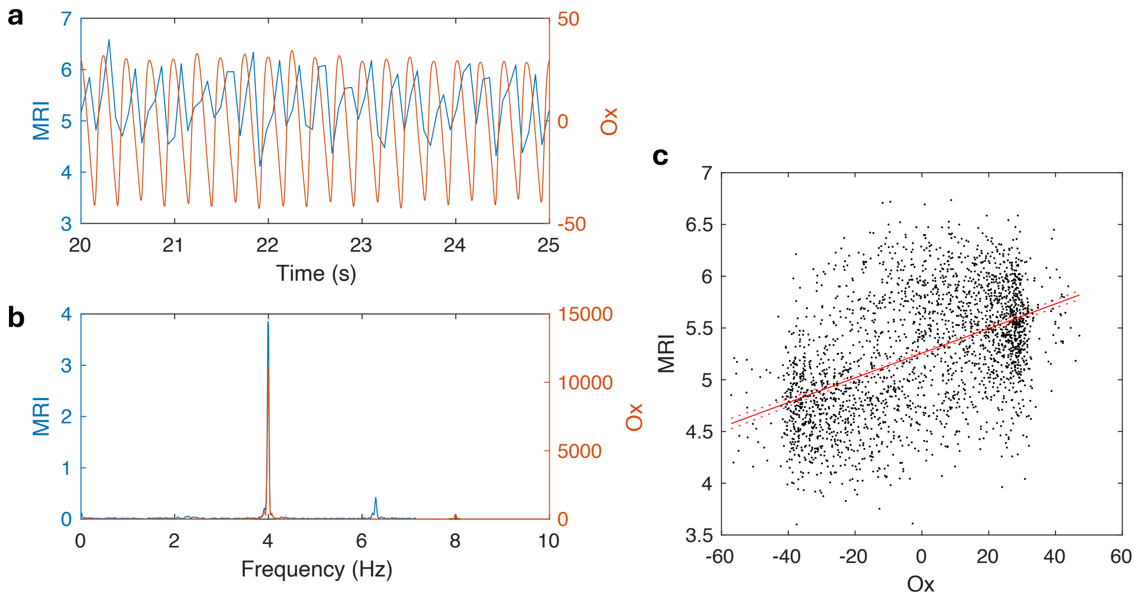

Supplementary Fig. 8. **Comparison of pulsation measured by MRI and pulse oximetry.** **a)** The pulsatile signal change measured by graident-echo MRI at the LHiA followed the pulsation measured by a pulse oximeter (Ox) positioned at the tail artery. **b)** The spectra of MRI and Ox signals show the same primary frequency peak corresponds to the heart rate. **c)** The amplitude changes correlated well ($r = 0.49$, $p < 6.45 \times 10^{-154}$).

Supplementary Table 1. Comparison of R^2 values of 4 different linear models for predicting hippocampal glymphatic signal kinetics based on hippocampal BOLD-CSF coupling and/or pulsation of the lateral hippocampal artery (LHiA). The following 4 models were compared:
Model 1: BOLD-CSF coupling only;
Model 2: arterial pulsation only;
Model 3: BOLD-CSF coupling and pulsation without their interaction;
Model 4: BOLD-CSF coupling, pulsation and their interaction.
The number in the parenthesis in the last column represents the additional R^2 explained by the interaction term. The number in bold indicates the best model for explaining the kinetics.

	BOLD-CSF coupling only	*LHiA pulsation only*	*BOLD-CSF + LHiA pulsation*	*Adding interaction*
Gd AUC	0.08	0.01	0.41	**0.73** (0.32)
Arrival time	0.06	0.57	**0.96**	0.97 (0.01)
Time-to-peak	**0.43**	0.01	0.45	0.66 (0.21)
Influx rate	0.07	0.10	0.58	**0.98** (0.40)
Decay time	0.39	0.48	**0.81**	0.82 (0.01)
