# Supplementary material for: Cholinergic basal forebrain neurons regulate vascular dynamics and cerebrospinal fluid flux
Source: Nat Commun. 2025 Jun 23;16:5343. doi: 10.1038/s41467-025-60812-3 (PMC12185694; doi:10.1038/s41467-025-60812-3)
Supplement: Supplementary file 2 — Reporting Summary [file 41467_2025_60812_MOESM2_ESM.pdf]

nature portfolio

Corresponding author(s): Kai-Hsiang Chuang

Last updated by author(s): May 24, 2025

Reporting Summary

Nature Portfolio wishes to improve the reproducibility of the work that we publish. This form provides structure for consistency and transparency in reporting. For further information on Nature Portfolio policies, see our Editorial Policies and the Editorial Policy Checklist.

Statistics

For all statistical analyses, confirm that the following items are present in the figure legend, table legend, main text, or Methods section.

n/a	Confirmed	
☐	☒	The exact sample size (n) for each experimental group/condition, given as a discrete number and unit of measurement
☐	☒	A statement on whether measurements were taken from distinct samples or whether the same sample was measured repeatedly
☐	☒	The statistical test(s) used AND whether they are one- or two-sided<br>*Only common tests should be described solely by name; describe more complex techniques in the Methods section.*
☒	☐	A description of all covariates tested
☐	☒	A description of any assumptions or corrections, such as tests of normality and adjustment for multiple comparisons
☐	☒	A full description of the statistical parameters including central tendency (e.g. means) or other basic estimates (e.g. regression coefficient) AND variation (e.g. standard deviation) or associated estimates of uncertainty (e.g. confidence intervals)
☐	☒	For null hypothesis testing, the test statistic (e.g. F, t, r) with confidence intervals, effect sizes, degrees of freedom and P value noted<br>*Give P values as exact values whenever suitable.*
☒	☐	For Bayesian analysis, information on the choice of priors and Markov chain Monte Carlo settings
☒	☐	For hierarchical and complex designs, identification of the appropriate level for tests and full reporting of outcomes
☐	☒	Estimates of effect sizes (e.g. Cohen's d, Pearson's r), indicating how they were calculated

Our web collection on statistics for biologists contains articles on many of the points above.

Software and code

Policy information about availability of computer code

Data collection	Human MRI and PET data were acquired on Siemens mMR scanner with standard protocols. Animal MRI data were acquired using Paravision 6.0.1 (Bruker GmbH) with standard and custom pulse sequences. The custom sequence was described in Lee,et al. NeuroImage 2019. The code for this custom pulse sequence is available from the corresponding author. The AMBMC mouse brain template can be downloaded from https://Imaging.org.au/AMBMC/AMBMC. The human brain Automated Anatomical Labeling (AAL) atlas can be downloaded from https://www.oxcns.org/aal3.html.
Data analysis	Data analysis were conducted using MATLAB (Mathworks Inc), Prism (GraphPad Inc) and open source software, including: FSL (https://www.fmrib.ox.ac.uk/fsl), AFNI (https://afni.nimh.nih.gov/), ANTs (http://stnava.github.io/ANTs/), and 3D-PCNN (https://sites.google.com/site/chuanglab/software/3d-pcnn).

For manuscripts utilizing custom algorithms or software that are central to the research but not yet described in published literature, software must be made available to editors and reviewers. We strongly encourage code deposition in a community repository (e.g. GitHub). See the Nature Portfolio guidelines for submitting code & software for further information.

Data

Policy information about availability of data

All manuscripts must include a data availability statement. This statement should provide the following information, where applicable:

- Accession codes, unique identifiers, or web links for publicly available datasets
- A description of any restrictions on data availability
- For clinical datasets or third party data, please ensure that the statement adheres to our policy

Source data for quantifications shown in all graphs plotted in figures and Extended Data figures are available in the online version of the paper. The raw data acquired in this study are also available from the corresponding authors upon reasonable request.

Research involving human participants, their data, or biological material

Policy information about studies with human participants or human data. See also policy information about sex, gender (identity/presentation), and sexual orientation and race, ethnicity and racism.

Reporting on sex and gender	Human data were collected from a well-balanced sample of females and males. Due to the relatively small sample size, sex was not considered as a primary variable, and sex- and gender-based analyses were not performed. The study was not powered to detect sex-related differences, and sex was not a central focus of the research objectives.
Reporting on race, ethnicity, or other socially relevant groupings	Data on race, ethnicity, or other socially relevant groupings were not collected, as they were not relevant to the research objectives.
Population characteristics	Participants were aged 55 years or older and included both males and females. Individuals with mild Alzheimer's disease (Mini-Mental State Examination [MMSE] ≥ 24) eligible for acetylcholinesterase inhibitor treatment were recruited from a memory clinic. Control participants were recruited from the same clinic, spouses of non-enrolled patients, or independent volunteers. All participants were fluent in English and from diverse cultural and linguistic backgrounds.
Recruitment	Participants were recruited from a single site, metropolitan hospital memory clinic at The Prince Charles Hospital (TPCH, Brisbane, Australia). All cognitively impaired participants met the Petersen criteria for mild cognitive impairment (MCI) including memory complaint, normal activities of daily living, normal general cognitive function with Mini-Mental State Examination (MMSE) ≥ 24, abnormal memory for age and lack of dementia (Petersen et al., 1999). Age-matched healthy control participants were primarily the spouses of patients from the memory clinic who have not been recruited into this study, in addition to other independent research volunteers. A flyer will be available at the memory clinic (approved by the TPCH HREC) outlining the nature and purpose of the study. Each group will provide informed written consent to participate in this study using separate consent forms.
Ethics oversight	Ethics approval was granted for the study by The Prince Charles Hospital (TPCH, Brisbane, Australia) Human Research Ethics Committee.

Note that full information on the approval of the study protocol must also be provided in the manuscript.

Field-specific reporting

Please select the one below that is the best fit for your research. If you are not sure, read the appropriate sections before making your selection.

[x] Life sciences   [ ] Behavioural & social sciences   [ ] Ecological, evolutionary & environmental sciences

For a reference copy of the document with all sections, see nature.com/documents/nr-reporting-summary-flat.pdf

Life sciences study design

All studies must disclose on these points even when the disclosure is negative.

Sample size	The sample size was informed by longitudinal amyloid accumulation data from the Australian Imaging, Biomarkers and Lifestyle (AIBL) study. Based on AIBL findings, a sample of 50 participants with MCI or mild Alzheimer's disease would be required to detect a statistically significant change in cortical amyloid load over 18 months (power = 90%, $p < 0.05$). Given the comparable sensitivity of [18F]-FEOBV PET for detecting cholinergic alterations in relation to amyloid burden, a similar sample size was considered appropriate for this study. However, recruitment was significantly disrupted due to COVID, and the target sample size could not be achieved. Therefore no statistical method was used to predetermine sample size for this study.
Data exclusions	Human imaging data that showed excessive motion were excluded.<br>Animal imaging data that did not show Gd contrast enhanced signal change in the glymphatic imaging, or detectable pulsatile signal change in the arterial pulsation imaging were excluded from the data analysis.

Replication	Human experiments were analyzed by combining all participants together and by separating them into MCI and control groups to assess whether the findings were biased by a sub-group.<br>Animal experiments were conducted in at least two separate batches of animals to ensure that the findings are not biased by batch to batch difference.
Randomization	Animals were randomly assigned as Lesion or Sham control group, or as DPZ treatment or Veh control group.
Blinding	Data acquisition, postprocessing and analysis were performed blind to the experimental group.

Reporting for specific materials, systems and methods

We require information from authors about some types of materials, experimental systems and methods used in many studies. Here, indicate whether each material, system or method listed is relevant to your study. If you are not sure if a list item applies to your research, read the appropriate section before selecting a response.

Materials & experimental systems

n/a	Involved in the study
☐	☒ Antibodies
☒	☐ Eukaryotic cell lines
☒	☐ Palaeontology and archaeology
☐	☒ Animals and other organisms
☒	☐ Clinical data
☒	☐ Dual use research of concern
☒	☐ Plants

Methods

n/a	Involved in the study
☒	☐ ChIP-seq
☒	☐ Flow cytometry
☐	☒ MRI-based neuroimaging

Antibodies

Antibodies used	Primary: goat anti-p75 antibody (AF1157, R&D Systems), mouse anti-CD68 antibody (MCA1957, Bio-Rad). anti-goat IgG Alexa Fluor 488 secondary antibody (A11055, Thermo Fisher Scientific) and DAPI (D9542, Sigma-Aldrich).
Validation	Goat anti-p75 antibody detects mouse NGFR/TNFRSF16 in direct ELISAs and Western blots. Reference: Saadipour et al, Molecular and Cellular Neuroscience 2019 https://pubmed.ncbi.nlm.nih.gov/31422108/.<br>Rat anti mouse CD68 antibody, clone FA-11, has been used in many mouse models for the identification of CD68 in immunohistochemical assays, using both frozen and paraffin-embedded tissues. Reference: Masaki et al. Nephrol Dial Transplant 2003 https://pubmed.ncbi.nlm.nih.gov/12480978/.

Animals and other research organisms

Policy information about studies involving animals; ARRIVE guidelines recommended for reporting animal research, and Sex and Gender in Research

Laboratory animals	Adult C57BL/6 mice were purchased from the Animal Resources Centre, WA, Australia. The animal was kept on a 12 h-12 h light-dark cycle at 20-22 degree C and 40-60% humidity. Animals were housed up to five per cage and were provided ad libitum access to water and food. Experiments were conducted at 13-16 weeks old.
Wild animals	none
Reporting on sex	Only female mice were used in this study to be consistent with our previous study (Zhou et al, Neurobiol. Aging 117: 24–32, 2022), in which female mice show stronger and earlier amyloid and tau pathological phenotypes than male.<br>It is well established that no sex difference in glymphatic function and cholinergic lesion effects has been found in mice. See:<br>Giannetto, M. et al. Biological sex does not predict glymphatic influx in healthy young, middle aged or old mice. Sci. Rep. 10, 16073 (2020).<br>Sherren, N. et al. Neural and behavioral effects of intracranial 192 IgG-saporin in neonatal rats: sexually dimorphic effects? Dev. Brain Res. 114, 49–62 (1999).<br>Qian, L. et al. Cholinergic basal forebrain degeneration due to sleep-disordered breathing exacerbates pathology in a mouse model of Alzheimer's disease. Nat. Commun. 13, 6543 (2022).<br><br>Yet, estrogen effect on mouse brain vascular dynamics remains to be elucidated. Differential immune response caused by lesion may also affect the glymphatic system and will need further investigation.
Field-collected samples	None
Ethics oversight	All animal use and experimental procedure were conducted following the Australian Code of Practice for the Care and Use of Animals for Scientific Purposes by National Health and Medical Research Council, and approved by the Animal Ethics Committee of the University af Queensland.

Note that full information on the approval of the study protocol must also be provided in the manuscript.

Plants

Seed stocks

none

Novel plant genotypes

Describe the methods by which all novel plant genotypes were produced. This includes those generated by transgenic approaches, gene editing, chemical/radiation-based mutagenesis and hybridization. For transgenic lines, describe the transformation method, the number of independent lines analyzed and the generation upon which experiments were performed. For gene-edited lines, describe the editor used, the endogenous sequence targeted for editing, the targeting guide RNA sequence (if applicable) and how the editor was applied.

Authentication

Describe any authentication procedures for each seed stock used or novel genotype generated. Describe any experiments used to assess the effect of a mutation and, where applicable, how potential secondary effects (e.g. second site T-DNA insertions, mosiacism, off-target gene editing) were examined.

Magnetic resonance imaging

Experimental design

Design type

Resting state.

Design specifications

No task fMRI.

Behavioral performance measures

Within 2 weeks of enrollment, cognitive assessments for the participants were conducted by an experienced neuropsychologist. The neuropsychological battery of this study were divided into four cognitive domains: memory (including Rey Auditory Verbal Learning Test – short delay and long delay and Wechsler Memory Scale - Visual Reproduction I and II), executive function (including Trail-Making Test B, Controlled Oral Word Association Test, Wechsler Adult Intelligence Scale - Digit Span Backwards), attention (including Trail-Making Test A and Victoria Stroop Test) and language (including Boston Naming Test and Semantic Fluency Test).

Acquisition

Imaging type(s)

Structural T1 (or T2)-weighted MRI, Resting-state BOLD fMRI, Arterial Spin Labeling MRI, Contrast-enhanced T1-weighted MRI, time-of-flight angiography.

Field strength

3T for human; 9.4T for animal

Sequence & imaging parameters

Human:
T1-weighted 3D MPRAGE image was acquired with TR = 2.3 s, echo time (TE) = 2.26 ms, inversion time = 0.9 s, flip angle = 8o, 1 mm isotropic resolution, and matrix 256 x 240 x 192. A T2-weighted fluid attention inversion recovery (FLAIR) image was acquired with TR = 5 s, TE = 386 ms, flip angle = 120o, 1 mm isotropic resolution, and matrix 256 x 256 x 160. Resting-state fMRI was conducted by 2D gradient-echo echo-planar imaging (EPI) with TR = 2.68 s, TE = 30 ms, flip angle = 90o, 3 mm isotropic resolution, matrix size 72 x 72 x 42, and 446 repetitions.

Animal:
Structural T2-weighted MRI scans of 0.1x0.1x0.3 mm3 resolution, field of view (FOV) = 19.2 x 19.2 x 16.8 mm3 was acquired using a 2D fast spin-echo with TR/TE = 5500/40 ms and five averages. Resting-state fMRI was conducted using a multiband gradient-echo EPI with TR = 300 ms, TE = 15 ms, 16 slices with 4 bands, resolution = 0.3 x 0.3 x 0.6 mm3, and 2000 repetitions. Arterial pulsation was measured by a single-shot gradient-echo EPI of 2 horizontal slices with thickness = 0.5 mm, 2.5 mm gap, in-plane resolution = 0.2 x 0.2 mm2, FOV = 19.2 x 12.8 mm, matrix size = 96 x 64, TR/TE = 70/14.15 ms, and flip angle = 90 degree and 2500 time frames. Pseudo-continuous arterial spin labelling sequence with labelling time = 3 s, post-labeling delay = 450 ms, and spin-echo echo planar imaging (EPI) of TR/TE=4414.68/19.36 ms and resolution = 0.3x0.3x0.6 mm3, and 74 pairs of label and control images. T1 map with TR=10 s and inversion times = 30, 50, 83, 138, 229, 380, 632, 1049, 1744, 2897, 4814 and 8000 ms was acquired for CBF quantification. Contrast-enhanced 3D T1-weighted fast low angle shot (FLASH) MRI (TR/TE = 21/2.66ms, flip angle = 20o, matrix = 192 x 128 x 80, 0.1 mm isotropic resolution, and 40 or 50 repetitions. Angiography was acquired using a time-of-flight (ToF) sequence with TR/TE = 17/3 ms, flip angle = 20o, slice number = 80, thickness = 0.35 mm, FOV = 20x20 mm2, matrix size = 320x320, and in-plane resolution = 0.0625 x 0.0625 mm2.

Area of acquisition

Brain.

Diffusion MRI ☐ Used ☒ Not used

Preprocessing

Preprocessing software

AFNI, FSL.

Normalization

Nonlinear transformed using ANTs.

Normalization template	MNI human brain template and AMBMC mouse brain template
Noise and artifact removal	Resting-state fMRI included despike, motion correction, nuisance regression and bandpass filter.
Volume censoring	None.

Statistical modeling & inference

Model type and settings	None.
Effect(s) tested	None.

Specify type of analysis: ☐ Whole brain ☐ ROI-based ☒ Both

Anatomical location(s)	Human: cortex and basal forebrain.<br>Animal: cortex and hippocampus.

Statistic type for inference (See Eklund et al. 2016)	Pearson's or Spearman correlation.<br>Two-sample t-test.
Correction	FWE correction using AFNI 3DClustSim in voxel-wise mapping.

Models & analysis

n/a	Involved in the study
☒	☐ Functional and/or effective connectivity
☒	☐ Graph analysis
☒	☐ Multivariate modeling or predictive analysis
